# The interaction of cytoplasmic poly(A)-binding protein with eukaryotic initiation factor 4G suppresses nonsense-mediated mRNA decay

**DOI:** 10.1261/rna.044933.114

**Published:** 2014-10

**Authors:** Tobias Fatscher, Volker Boehm, Benjamin Weiche, Niels H. Gehring

**Affiliations:** 1 Institute for Genetics, University of Cologne, 50674 Cologne, Germany

**Keywords:** PABPC1, eIF4G, NMD, ribosome recycling, translation termination

## Abstract

Nonsense-mediated mRNA decay (NMD) eliminates different classes of mRNA substrates including transcripts with long 3′ UTRs. Current models of NMD suggest that the long physical distance between the poly(A) tail and the termination codon reduces the interaction between cytoplasmic poly(A)-binding protein (PABPC1) and the eukaryotic release factor 3a (eRF3a) during translation termination. In the absence of PABPC1 binding, eRF3a recruits the NMD factor UPF1 to the terminating ribosome, triggering mRNA degradation. Here, we have used the MS2 tethering system to investigate the suppression of NMD by PABPC1. We show that tethering of PABPC1 between the termination codon and a long 3′ UTR specifically inhibits NMD-mediated mRNA degradation. Contrary to the current model, tethered PABPC1 mutants unable to interact with eRF3a still efficiently suppress NMD. We find that the interaction of PABPC1 with eukaryotic initiation factor 4G (eIF4G), which mediates the circularization of mRNAs, is essential for NMD inhibition by tethered PABPC1. Furthermore, recruiting either eRF3a or eIF4G in proximity to an upstream termination codon antagonizes NMD. While tethering of an eRF3a mutant unable to interact with PABPC1 fails to suppress NMD, tethered eIF4G inhibits NMD in a PABPC1-independent manner, indicating a sequential arrangement of NMD antagonizing factors. In conclusion, our results establish a previously unrecognized link between translation termination, mRNA circularization, and NMD suppression, thereby suggesting a revised model for the activation of NMD at termination codons upstream of long 3′ UTR.

## INTRODUCTION

NMD represents a surveillance mechanism that removes transcripts with premature translation termination codons (PTCs) from eukaryotic cells (Chang et al. 2007; Rebbapragada and Lykke-Andersen 2009; Nicholson et al. 2010). The core NMD factors are present in all eukaryotes and their activity prevents the synthesis of C-terminally truncated proteins with potentially dominant negative effects (Bhuvanagiri et al. 2010). Furthermore, NMD directly or indirectly regulates the expression of many physiological mRNAs, although only some of them contain PTCs, for example, as a result of alternative splicing or upstream open reading frames (Yepiskoposyan et al. 2011; Tani et al. 2012). The function of NMD as a general regulator of gene expression explains why NMD factors are essential for normal animal development (Hwang and Maquat 2011).

In human cells, NMD is efficiently activated when at least one intron is located >50 nt downstream from the termination codon (Thermann et al. 1998; Zhang et al. 1998). During splicing in the nucleus, exon–exon junctions are marked by exon-junction complexes (EJCs), which serve as NMD-activating signals during translation in the cytoplasm. In addition to the aforementioned EJC-dependent NMD, an alternative EJC-independent NMD pathway targets mRNAs with a long 3′ UTR (Eberle et al. 2008; Singh et al. 2008; Yepiskoposyan et al. 2011). During eukaryotic translation termination, the interaction of PABP (in humans PABPC1) with the ribosome-bound eRF3 (in humans eRF3a) stimulates polypeptide release and the subsequent recycling of ribosomes (Hoshino et al. 1999; Uchida et al. 2002). However, when the interaction of PABPC1 with eRF3a is reduced by an unusually long 3′ UTR, UPF1 binds to eRF3a and activates NMD (Singh et al. 2008). Hence, tethering of PABPC1 in the proximity of a termination codon can inhibit NMD by simulating the presence of a poly(A) tail (Amrani et al. 2004; Behm-Ansmant et al. 2007; Eberle et al. 2008; Silva et al. 2008; Singh et al. 2008).

In the case of premature translation termination, UPF1 is phosphorylated by SMG1 within its extended N- and C-terminal regions (Kashima et al. 2006). UPF2 binds directly to the C-terminal part of SMG1 and stimulates the phosphorylation of UPF1 (Kashima et al. 2006; Clerici et al. 2013). Phosphorylated UPF1 recruits the homologous proteins SMG5/SMG7 and SMG6, leading to the degradation of the target mRNA (Okada-Katsuhata et al. 2012). While the exonucleolytic decay is coordinated by the SMG5-SMG7 heterodimer (Loh et al. 2013), the endonucleolytic cleavage of NMD targets is mediated by the C-terminal PIN (PilT N terminus) domain of SMG6 (Glavan et al. 2006; Huntzinger et al. 2008; Eberle et al. 2009).

A large distance between the poly(A) tail and the termination codon promotes NMD. Therefore, long 3′ UTRs represent an NMD activating characteristic of endogenous NMD targets (Eberle et al. 2008; Singh et al. 2008; Yepiskoposyan et al. 2011). In view of the many endogenous mRNAs that are potentially regulated by this pathway, it is important to elucidate the molecular mechanism of NMD suppression by PABPC1. Using the MS2 tethering system, we have investigated which molecular interactions of PABPC1 are required to inhibit NMD of a reporter mRNA with a long 3′ UTR. We find that tethered PABPC1 suppresses NMD induced by a long 3′ UTR. Moreover, PABPC1 does not require the interaction with eRF3a to retain its NMD suppressing activity. In contrast, a mutant of PABPC1 unable to bind eIF4G does not inhibit NMD. Furthermore, tethered eIF4G or eRF3a suppress NMD as well. Our observations suggest a tight coupling between mRNA circularization via eIF4G and NMD suppression.

## RESULTS

### Reporter-bound PABPC1 increases long 3′ UTR-containing mRNA levels

**FIGURE 1.**
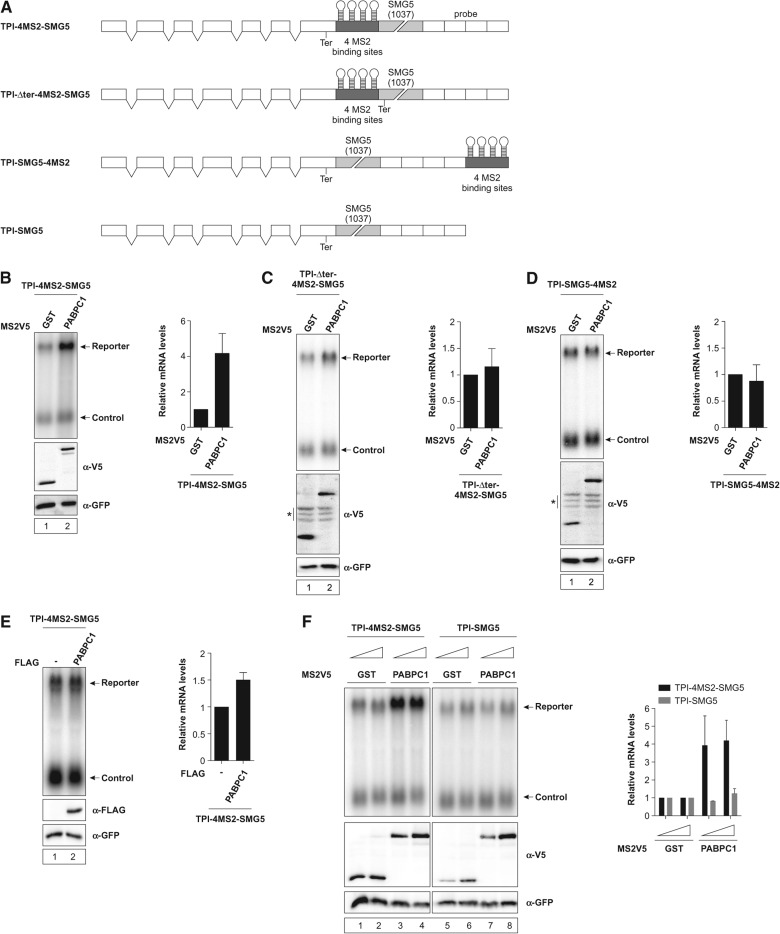
Tethering PABPC1 to a reporter mRNA containing a long 3′ UTR increases mRNA abundance. (*A*) Schematic representation of the triosephosphate isomerase (TPI) reporter constructs. White boxes depict exons, introns are shown as two connecting black lines, and Northern probe binding sites as white boxes without intron lines. Gray boxes represent MS2-binding area with MS2-stem–loops shown in black. The SMG5 3′ UTR is depicted as a light-gray box and the length in nucleotides is shown in brackets. (*B*–*F*) Northern blot analysis of total RNA extracted from HeLa cells transfected with plasmids expressing the indicated TPI reporter mRNA and MS2V5- or FLAG-tagged fusion proteins. A β-globin construct was cotransfected as control. Protein expression was detected by immunoblotting with α-V5 or α-FLAG antibody. Cotransfected GFP served as a loading control. Asterisks indicate unspecific bands (*C*,*D*). mRNA levels were normalized to MS2V5-GST (*B*–*D*,*F*) or pCI-FLAG (*E*). Bars represent the mean values of mRNA levels±SD upon tethering MS2V5-GST or MS2V5-PABPC1 (*B*–*D*,*F*), or pCI-FLAG and FLAG-PABPC1 (*E*). Concentrations of MS2V5-tagged protein expressing plasmids were increased from 1 µg (*F*, lanes *1*,*3*,*5*,*7*) to 3 µg (*F*, lanes *2*,*4*,*6*,*8*).

PABPC1 plays a pivotal role in gene expression because it promotes mRNA circularization, facilitates ribosome recycling, and suppresses NMD at normal termination codons (Wells et al. 1998; Behm-Ansmant et al. 2007). To analyze NMD suppression by PABPC1, we used a reporter construct consisting of the triosephosphate-isomerase (TPI) open reading frame (ORF) to which we added the 3′ UTR of SMG5. The SMG5 mRNA has been previously shown to undergo NMD mediated by its long 3′ UTR (Singh et al. 2008) and owing to the presence of the SMG5 3′ UTR, the reporter mRNA is degraded (V Boehm, N Haberman, F Ottens, J Ule, and NH Gehring, in prep.). We inserted four MS2 binding sites downstream from the termination codon to enable tethering of MS2-fusion proteins to a position at the beginning of the 3′ UTR (TPI-4MS2-SMG5) (Fig. 1A). Upon coexpression (i.e., tethering) of MS2V5-tagged PABPC1, the levels of the reporter mRNA increased by a factor of four compared with the MS2V5-GST that served as negative control (Fig. 1B). This suggests that PABPC1 counteracts NMD of the reporter mRNA. To confirm that the observed increase in mRNA abundance is an NMD-specific effect and not due to general mRNA stabilization by PABPC1, we used two additional TPI control reporter constructs. In one construct, the termination codon was shifted downstream from the MS2 binding sites by deleting the original termination codon (TPI-Δter-4MS2-SMG5) (Fig. 1A). In the other construct, the four MS2-binding sites were moved to a position at the 3′ end of the 3′ UTR (TPI-SMG5-4MS2) (Fig. 1A). In both cases, tethering of PABPC1 only marginally changes the levels of the reporter mRNAs (Fig. 1C,D). To exclude possible *trans*-effects of PABPC1 expression, we coexpressed either FLAG-PABPC1 together with the TPI-4MS2-SMG5 reporter (Fig. 1E), or MS2V5-PABPC1 with a TPI-SMG5 reporter lacking MS2-binding sites (Fig. 1A,F). In both cases, we observed only slight increases in mRNA levels when PABPC1 was not directly bound. In summary, our results suggest that PABPC1 is able to antagonize NMD induced by the long 3′ UTR of the reporter mRNA when it is tethered in close proximity downstream from the termination codon.

### PABPC1 stabilizes mRNA by inhibition of NMD

**FIGURE 2.**
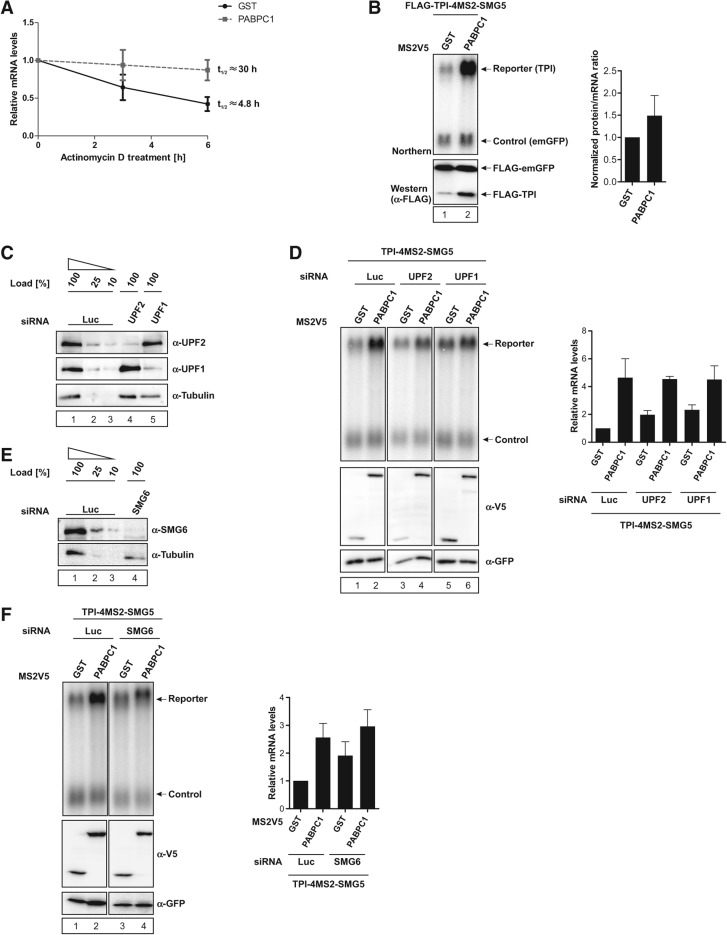
PABPC1 stabilizes reporter mRNA by suppressing NMD. (*A*) HeLa cells expressing reporter (TPI-4MS2-SMG5) and control mRNA, as well as MS2V5-tagged GST or PABPC1, were treated with Actinomycin D (5 µg/mL final concentration) for the indicated time prior to harvesting. Reporter mRNA levels were quantified by Northern blotting, normalized to control mRNA and GST control tethering, and plotted against time of Actinomycin D treatment. (*B*) Tethering of MS2V5-GST and -PABPC1 in HeLa cells cotransfected with N-terminally FLAG-tagged TPI-4MS2-SMG5 reporter and FLAG-tagged emGFP control expressing vectors. Both reporter and control mRNA contained heterologous binding sites in the 3′ UTR that enable the detection with the same Northern probe. Northern blot (*top*) and α-FLAG Western blot (*bottom*) analyses are shown. The signals for emGFP and TPI in Northern and Western blot experiments were quantified, normalized to the GST control lane, and the final ratio was calculated by normalizing protein expression levels to the respective mRNA expression levels. (*C*,*E*) siRNA-mediated knockdown of UPF2, UPF1, and SMG6. HeLa cells were transfected with siRNAs targeting UPF2, UPF1, SMG6, or Luciferase (negative control). The knockdown efficiency was assessed by immunoblotting with UPF2, UPF1, and SMG6-specific antibodies. Tubulin served as a loading control. (*D*,*F*) Northern blot analysis of TPI-4MS2-SMG5 reporter mRNA with β-globin mRNA as control in UPF2 (*D*, lanes *3*,*4*), UPF1 (*D*, lanes *5*,*6*), and SMG6 (*F*, lanes *3*,*4*) knockdown and control (*D*,*F*, lanes *1*,*2*) cells. HeLa cells were transfected with plasmids expressing MS2V5-GST and -PABPC1 proteins. Protein expression was detected by immunoblotting with a V5 antibody. GFP served as a loading control. mRNA levels were normalized to MS2V5-GST. Bars represent the mean values of mRNA levels ±SD upon tethering MS2V5-GST and -PABPC1 fusion proteins.

The previous experiments analyzed the steady-state levels of reporter mRNA. To confirm that tethered PABPC1 stabilizes the mRNA, we next determined the decay rates of the reporter mRNA upon transcriptional shutoff by Actinomycin D treatment. We observed a half-life of ∼4.8 h of the TPI-4MS2-SMG5 reporter construct when MS2V5-GST is tethered as a control (Fig. 2A). Upon PABPC1 tethering to the same reporter the mRNA is stabilized with a half-life of ∼30 h (Fig. 2A). This demonstrates that MS2V5-PABPC1 is in fact able to suppress NMD by preventing the degradation of the reporter mRNA construct. Because NMD is restricted to actively translated mRNAs (Thermann et al. 1998), we aimed to confirm that the observed effects are not caused by decreased translation rates of the reporter mRNAs. To this end, we used an N-terminally FLAG-tagged TPI-4MS2-SMG5 reporter construct, enabling us to measure translation efficiency by Western blotting and to correlate these effects with the mRNA levels detected by Northern blotting. FLAG-tagged emGFP was cotransfected as a loading control for both the mRNA as well as protein expression levels. Tethering MS2V5-PABPC1 to the FLAG-tagged TPI reporter mRNA led to a similar increase in FLAG-TPI protein and mRNA levels compared with the GST control (Fig. 2B, left), indicating that tethered PABPC1 does not change overall translation rates (Fig. 2B, right). We obtained similar results using a dual luciferase reporter system (data not shown). These results indicate that the NMD antagonizing effect of tethered PABPC1 is not caused by decreased translation efficiency but rather by protecting the mRNA from degradation.

Human NMD is executed by a core machinery including the central NMD factors UPF1 and UPF2. To confirm that PABPC1 inhibits the canonical NMD pathway, we used small-interfering RNAs (siRNAs) to deplete UPF1 and UPF2 in human cell culture. Numerous studies have shown that NMD is impaired in cells lacking either of these two factors (Mendell et al. 2002; Gatfield et al. 2003). UPF1 and UPF2 were reduced to ∼10% of regular expression levels by RNAi, as shown by immunoblotting (Fig. 2C). The levels of the reporter mRNAs were increased when UPF1 or UPF2 were depleted, confirming the inhibition of NMD by the transfected siRNAs (Fig. 2D, cf. lanes 1,3,5). Tethering of PABPC1 to the TPI-4MS2-SMG5 reporter mRNA increased reporter mRNA levels by a factor of more than four in control cells (Fig. 2D, lane 2), in line with our previous results. In contrast, tethering PABPC1 to the reporter only increased the mRNA levels by a factor of about two in both UPF2- and UPF1-knockdown cells (Fig. 2D, lanes 4,6). Notably, even though the change of mRNA levels by PABPC1 tethering was reduced in the UPF1 and UPF2 knockdown cells, the total levels of stabilized reporter mRNA under all three conditions reached a similar level (Fig. 2D, cf. lanes 2,4,6). These results indicate that PABPC1 acts as a strong suppressor in the canonical NMD pathway that involves the central NMD factors UPF1 and UPF2.

As described above, PABPC1 suppresses UPF1- and UPF2-dependent NMD. Several decay pathways act downstream from UPF1 to ensure efficient degradation of substrate mRNAs (Nicholson and Muhlemann 2010). We speculated that the NMD inhibiting effect of PABPC1 also impinges on the degradation phase of NMD. To test this hypothesis, we established the depletion of the NMD-specific endonuclease SMG6 by RNAi. In knockdown cells, the SMG6 protein levels were reduced to ∼10% of regular expression levels (Fig. 2E). Similar to our results in UPF1- and UPF2-depleted cells, tethered PABPC1 stabilized the mRNA levels by a factor of less than two in SMG6-depleted cells (Fig. 2F, cf. lanes 2,4), while stabilized reporter levels remained unchanged compared with control knockdown cells. Taken together with the results obtained so far, this shows that PABPC1 indeed inhibits NMD by preventing the degradation of the substrate mRNA.

### The interaction of PABPC1 and eRF3a is dispensable for NMD suppression

**FIGURE 3.**
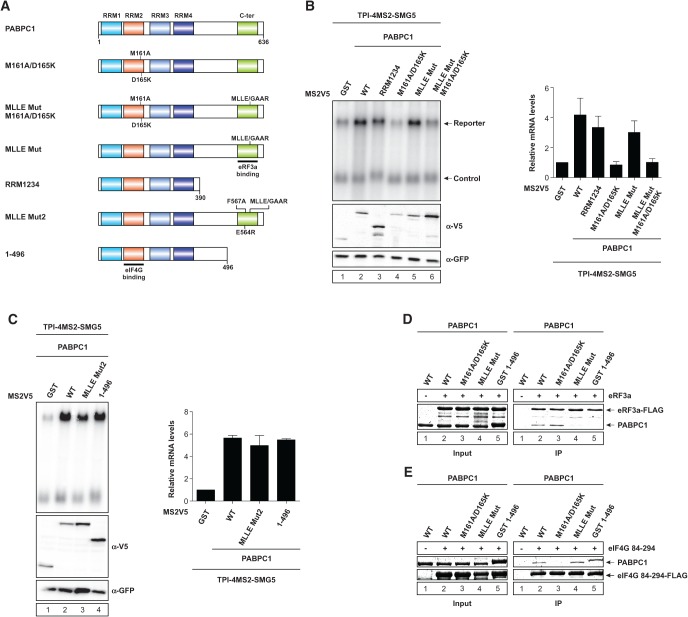
PABPC1 interaction with eIF4G, but not eRF3a, is essential for NMD suppression. (*A*) Schematic representation of PABPC1 domains and mutants. RNA recognition motifs (RRMs) and C-terminal region (C-ter) of PABPC1 are highlighted. Point mutations and binding sites are indicated. (*B*,*C*) HeLa cells were transfected with plasmids expressing the indicated MS2V5-tagged fusion proteins and the indicated TPI reporter mRNA. Northern blot analysis was performed and cotransfected β-globin mRNA construct served as control. Protein expression was detected by immunoblotting with a V5 antibody. GFP served as a loading control. mRNA levels were normalized to MS2V5-GST. Bars represent mean values of mRNA levels ±SD upon tethering of different MS2V5-tagged fusion proteins. (*D*,*E*) Pull-down assays of in vitro interaction studies using PABPC1 mutants and FLAG-tagged eRF3a or eIF4G 84-294. Proteins were visualized with Coomassie Brilliant Blue.

Current models of NMD suggest that PABPC1 competes with the NMD factor UPF1 for eRF3a binding (Singh et al. 2008). To more specifically elucidate which domains and interaction regions of PABPC1 are responsible for the NMD suppression effect observed in Figure 1, we generated six mutants of PABPC1 (Fig. 3A). PABPC1 interacts via its C-terminal MLLE domain with two PAM2 motifs present in the N terminus of eRF3a (Kozlov and Gehring 2010). We designed two mutants to impair this interaction: one consisting of RNA recognition motifs (RRMs) one to four of PABPC1 (PABPC1 RRM^1234^) (Fig. 3A), i.e., lacking the C-terminal domain; the second containing a mutation of the MLLE motif to GAAR (PABPC1 MLLE^Mut^) (Fig. 3A). The tethering assay shows that both PABPC1 RRM^1234^ and PABPC1 MLLE^Mut^ suppressed NMD to almost the same extent as PABPC1 (Fig. 3B, lanes 2,3,5). We further tested two additional PABPC1 mutants abrogating PAM2 binding, one containing the MLLE mutation with additional point mutations known to further abolish PAM2 motif binding (Kozlov et al. 2004) (PABPC1 MLLE^Mut2^) (Fig. 3A) and a longer version of RRM^1234^ including additional C-terminal amino acids (PABPC1 1-496) (Fig. 3A). Tethering either of these mutants to the TPI-4MS2-SMG5 reporter construct increases the mRNA abundance of the reporter to the same degree as PABPC1 (Fig. 3C). Notably, it was postulated that the direct binding of PABPC1 to eRF3a outcompetes the eRF3a-UPF1 interaction. However, our results suggest that this interaction, as well as other interactions involving the MLLE motif of PABPC1, is not strictly necessary for tethered PABPC1 to suppress NMD.

### PABPC1 unable to bind eIF4G fails to stabilize reporter mRNA

The RRM^1234^ region of PABPC1 contains RRM2, which binds to a short N-terminal motif within eIF4G (Safaee et al. 2012). This interaction is important for mRNA circularization and the efficient expression of polyadenylated mRNAs (Wells et al. 1998; Amrani et al. 2008). Guided by the molecular structure of the PABPC1-eIF4G-RNA ternary complex (Safaee et al. 2012), we introduced two point mutations into PABPC1, which abolish binding to eIF4G (PABPC1^M161A/D165K^) (Fig. 3A; Kahvejian et al. 2005). Strikingly, tethering of PABPC1^M161A/D165K^ to the reporter no longer suppressed NMD and mRNA levels remained unchanged (Fig. 3B, lane 4). A similar result was obtained with the PABPC1 MLLE^Mut M161A/D165K^ mutant that can neither interact with eIF4G nor eRF3a (Fig. 3B, lane 6). These results indicate that the interaction between PABPC1 and eIF4G is critical for the inhibition of NMD by tethered PABPC1.

To show that the results we have generated so far are indeed due to interactions or lack thereof between PABPC1 with eIF4G and eRF3a, we have performed in vitro interaction studies. Pull-down assays of C-terminally FLAG-tagged eRF3a with both of the PABPC1 MLLE^Mut^ and PABPC1 1–496 mutants show that they are no longer able to interact with eRF3a, whereas the wild-type as well as the PABPC1^M161A/D165K^ mutant are still able to be pulled down by eRF3a (Fig. 3D). The same experiment was performed with a shortened version of FLAG-tagged eIF4G (eIF4G 84–294) containing the PABPC1 binding site. In this experimental setup eIF4G 84–294 was no longer able to pull-down the PABPC1^M161A/D165K^ mutant (Fig. 3E). The wild-type as well as the PABPC1 MLLE^Mut^ and PABPC1 1–496 mutants were still able to interact with eIF4G 84–294 (Fig. 3E).

In summary, our results demonstrate that PABPC1 inhibits NMD when tethered upstream of an NMD-activating long 3′ UTR. Furthermore, binding of eIF4G but not eRF3a contributes to NMD suppression by tethered PABPC1. Interestingly, our findings suggest a previously unrecognized role of eIF4G-mediated mRNA circularization and ribosome recycling as modulators of NMD.

### EJC-dependent NMD is mostly unaffected by PABPC1

**FIGURE 4.**
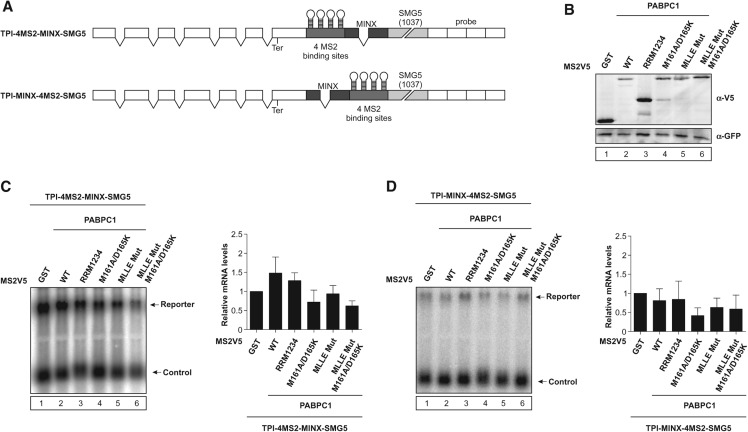
PABPC1 primarily functions as a suppressor in EJC-independent NMD. (*A*) Schematic representation of the TPI reporters as in Figure 1A. Dark-gray boxes indicate intron-containing MINX cassette. (*B*) Representative Western blot showing MS2V5-tagged protein expression, as analyzed by immunoblotting with a V5 antibody. (*C*,*D*) HeLa cells were transfected with plasmids expressing the indicated MS2V5-tagged fusion proteins and the indicated TPI reporter mRNA. Northern blot analysis was performed and cotransfected β-globin mRNA construct served as control. mRNA levels were normalized to MS2V5-GST. Bars represent mean values of mRNA levels ±SD upon tethering of different MS2V5-tagged fusion proteins.

So far, we have examined the suppression of NMD activated by the SMG5 3′ UTR. However, NMD of many nonsense-containing mRNAs occurs in a splicing-dependent manner and involves EJCs deposited at exon–exon junctions. Therefore, we wanted to analyze whether PABPC1 is able to antagonize EJC-dependent NMD. To this end, we constructed a reporter with an intron (MINX) downstream from the 4MS2-binding sites and upstream of the long 3′ UTR (TPI-4MS2-MINX-SMG5) (Fig. 4A). Splicing of the intron deposits an EJC that will activate NMD at the termination codon of the reporter mRNA. Strikingly, tethering of PABPC1 only weakly inhibited EJC-dependent NMD and slightly increased the MINX-containing mRNA levels by a factor of less than two (Fig. 4B,C, lane 2). In general, the inhibition of EJC-dependent NMD by PABPC1 variants was clearly reduced when compared with the inhibition of EJC-independent NMD (cf. Figs. 3B and 4C). Hence, the presence of an EJC appears to reduce the ability of PABPC1 to antagonize NMD, which explains why EJC-dependent NMD efficiently degrades mRNAs with short 3′ UTRs.

The weak inhibition of EJC-dependent NMD observed in Figure 4C was completely lost when we used a reporter construct, in which the MINX intron was inserted between the tethering sites and the termination codon (TPI-MINX-4MS2-SMG5) (Fig. 4A). Neither PABPC1 nor any of its mutants were able to increase mRNA levels of this reporter (Fig. 4B,D), which demonstrates that PABPC1 cannot antagonize the activation of NMD in the presence of an upstream EJC. Since an EJC in close proximity downstream from tethered PABPC1 is able to decrease the NMD suppression by PABPC1, we conclude that PABPC1 mainly regulates the EJC-independent NMD of mRNAs with long 3′ UTRs.

### Tethered eRF3a relies on interaction with PABPC1 to antagonize NMD

**FIGURE 5.**
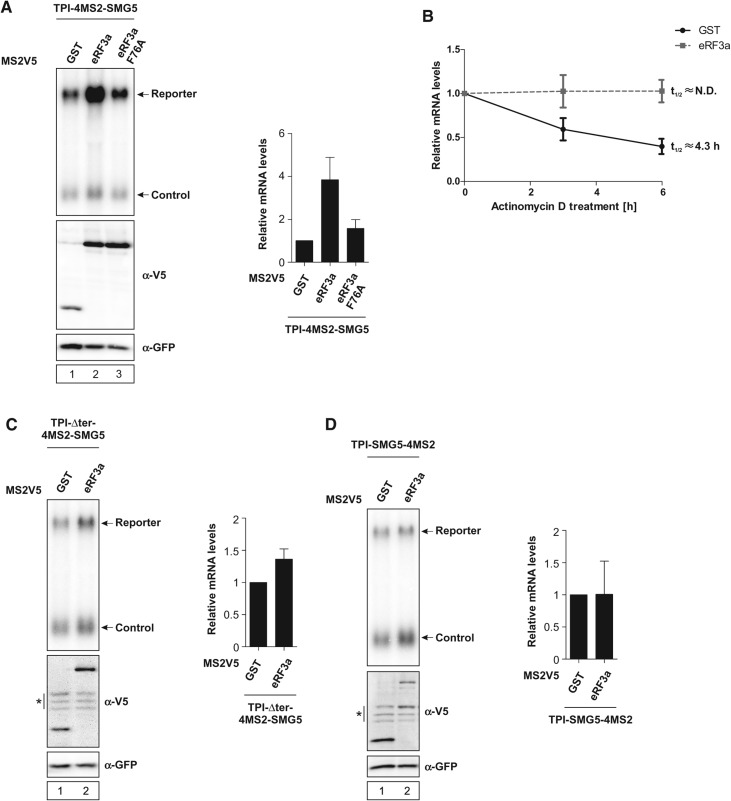
Tethered eRF3a unable to interact with PABPC1 fails to stabilize reporter mRNA levels. (*A*,*C*,*D*) HeLa cells were transfected with plasmids expressing the indicated MS2V5-tagged fusion proteins and the indicated TPI reporter mRNA. Northern blot analysis was performed and cotransfected β-globin mRNA construct served as control. Protein expression was detected by immunoblotting with a V5 antibody. GFP served as a loading control. Asterisks indicate unspecific bands (*C*,*D*). mRNA levels were normalized to MS2V5-GST. Bars represent mean values of mRNA levels ±SD upon tethering of different MS2V5-tagged fusion proteins. (*B*) HeLa cells expressing MS2V5-tagged GST or eRF3a were treated with Actinomycin D for the indicated time. mRNA levels were quantified after Northern blot analysis and plotted as described in Figure 2.

To gain further insight into the role of eRF3a in NMD suppression, we tethered eRF3a to the TPI-4MS2-SMG5 reporter construct, which led to increased reporter mRNA abundance by a factor of four, demonstrating eRF3a's ability to suppress NMD similar to PABPC1 (Fig. 5A, lane 2). To investigate the importance of the interaction between eRF3a and PABPC1 in NMD suppression, we used an eRF3a mutant carrying a point mutation (eRF3a F76A), which is essential for binding to PABPC1 (Kononenko et al. 2010; Kozlov and Gehring 2010; Osawa et al. 2012). Compared with eRF3a WT, the eRF3a F76A mutant has lost its NMD suppressing activity (Fig. 5A, lane 3).

To ensure that the observed effects are in fact due to NMD suppression, we determined mRNA half-life when tethering eRF3a. Similar to PABPC1, eRF3a clearly increased mRNA half-life, confirming that it specifically suppresses NMD and stabilizes the reporter mRNA (Fig. 5B). Furthermore, the specificity of the effects observed for tethered eRF3a were confirmed with both control reporter constructs described in Figure 1. Tethering eRF3a to either reporter did only marginally change mRNA abundance (Fig. 5C,D).

In summary, these results demonstrate that the interaction with PABPC1 is essential for eRF3a to suppress NMD initiated by a long 3′ UTR.

### Recruitment of eIF4G increases mRNA abundance independently of interaction with PABPC1

**FIGURE 6.**
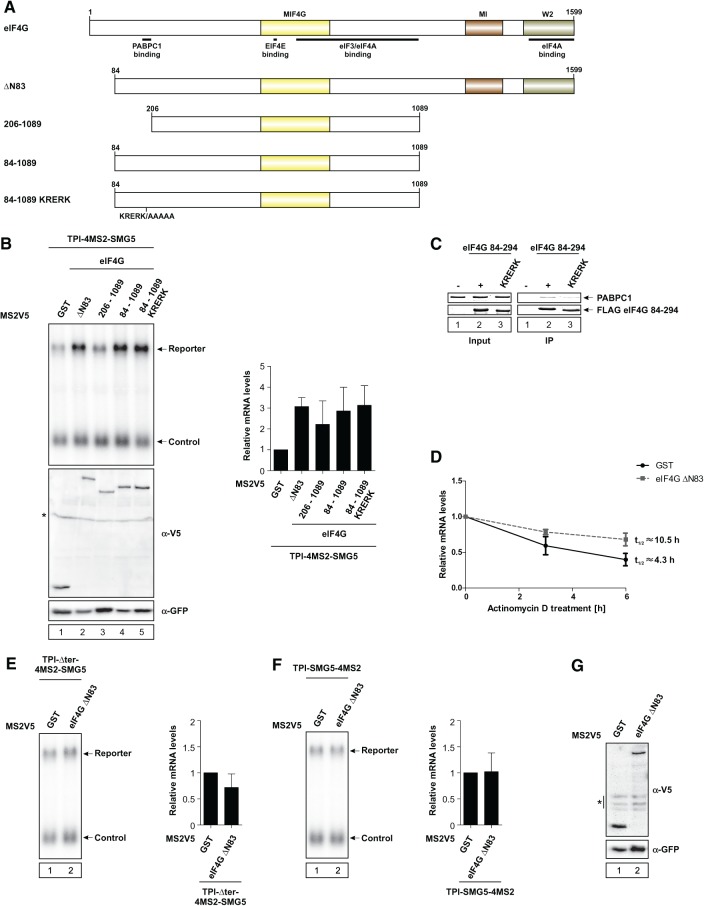
The interaction with PABPC1 is dispensable for NMD suppression by tethered eIF4G. (*A*) Illustration of eIF4G domain architecture and mutants. MIF4G, MI, and W2 domains are highlighted. Point mutations and binding sites are indicated. (*B*,*E*,*F*) HeLa cells were transfected with plasmids expressing the indicated MS2V5-tagged fusion proteins and the indicated TPI reporter mRNA. Northern blot analysis was performed and cotransfected β-globin mRNA construct served as control. Protein expression was detected by immunoblotting with a V5 antibody. GFP served as a loading control. Asterisk indicates an unspecific band (*B*). mRNA levels were normalized to MS2V5-GST. Bars represent mean values of mRNA levels ±SD upon tethering of different MS2V5-tagged fusion proteins. (*C*) Pull-down assays of in vitro interaction studies using PABPC1 and FLAG-tagged eIF4G 84-294 WT or KRERK mutant. Proteins were visualized with Coomassie Brilliant Blue. (*D*) HeLa cells expressing MS2V5-tagged GST or eIF4G ΔN83 were treated with Actinomycin D for the indicated time. mRNA levels were quantified after Northern blot analysis and plotted as described in Figure 2. (*G*) Representative Western blot showing MS2V5-tagged protein expression, as analyzed by immunoblotting with a V5 antibody. GFP served as a loading control. Asterisk indicates an unspecific band.

Next, we aimed to further investigate NMD suppression by eIF4G and the role of the interaction between PABPC1 and eIF4G. An N-terminally truncated version of eIF4G (eIF4G ΔN83) was used to analyze the function of eIF4G in NMD suppression (Fig. 6A).

Similar to PABPC1, we find that eIF4G ΔN83 is able to antagonize NMD, increasing the levels of the reporter mRNA by a factor of three (Fig. 6B, lane 2). To further elucidate the role of the interaction between eIF4G and PABPC1 in NMD suppression, we tethered different deletion mutants of eIF4G to the reporter construct (Fig. 6A). A shortened version of eIF4G ranging from amino acids 84 to 1089 (eIF4G 84–1089) (Fig. 6A) was still able to fully suppress NMD, which is indicated by an increase in mRNA reporter levels of a factor of three (Fig. 6B, lane 4). Surprisingly, a shortened version of eIF4G carrying a mutation of the KRERK motif at position 187–191 (eIF4G 84–1089 KRERK) (Fig. 6A), which is responsible for PABPC1 binding (Wakiyama et al. 2000), efficiently antagonized NMD (Fig. 6B, lane 5). Although the eIF4G KRERK mutant has previously been shown to abrogate binding between eIF4G and PABPC1 (Wakiyama et al. 2000), our pull-down assay indicates that the eIF4G KRERK mutant is still able to interact with PABPC1, albeit to a lesser degree (Fig. 6C). Hence, the full NMD suppression activity of tethered eIF4G 84–1089 KRERK might be due to partially retained PABPC1-binding. However, an eIF4G deletion mutant lacking the PABPC1-binding domain (eIF4G 206–1089) (Fig. 6A) had a slightly decreased NMD suppression rate, but was still able to increase mRNA abundance by a factor of two (Fig. 6B, lane 3). These results indicate that the interaction of eIF4G with PABPC1 is favorable but not absolutely necessary for eIF4G's ability to suppress NMD.

mRNA half-life was measured to ensure that the observed effects are in fact due to NMD suppression. Similar to PABPC1 and eRF3a, but less efficiently, eIF4G ΔN83 increased mRNA half-life, confirming that it specifically suppresses NMD and stabilizes the reporter mRNA (Fig. 6D). Notably, the low expression levels of the eIF4G constructs may account for the less prominent stabilization effects compared with tethered PABPC1 and eRF3a.

We confirmed the specificity of the effects observed for tethered eIF4G with both control reporter constructs described in Figure 1. Tethering eIF4G to either reporter only marginally changed mRNA abundance (Fig. 6E–G).

The results presented here indicate that suppression of NMD by tethered eIF4G does not strictly require binding to PABPC1, albeit this interaction might enhance the function of eIF4G.

## DISCUSSION

While the process and the function of NMD has been elucidated in great detail and many factors and determinants that activate NMD are known, the mechanism of NMD suppression is far less understood. In this study, we report that the inhibition of EJC-independent NMD requires the interaction of PABPC1 with the initiation factor eIF4G. We further show that PABPC1 suppresses a SMG6-dependent canonical NMD pathway involving the central NMD factors UPF1 and UPF2. Our data suggest that the molecular processes of translation termination and mRNA circularization impinge on the activation of NMD.

The NMD-inhibitory function of PABPs has been observed in different eukaryotic organisms, such as yeast (Amrani et al. 2004), fly (Behm-Ansmant et al. 2007), and humans (Eberle et al. 2008; Ivanov et al. 2008; Silva et al. 2008; Singh et al. 2008). However, the NMD-specific function of PABP has proven to be difficult to study separately from its other important functions in mRNA translation and stabilization. Furthermore, human NMD is characterized by different signals for NMD activation and multiple pathways for degradation, contrary to many other organisms that use one main NMD mechanism (Hwang and Maquat 2011).

Human NMD occurs either in an EJC-dependent manner, when a PTC is located upstream of the last intron position, or independently of EJCs at termination codons that are followed by long 3′ UTRs (Schweingruber et al. 2013). We used a reporter mRNA with a long 3′ UTR to study the suppression of NMD by PABPC1. Our results demonstrate that tethering PABPC1 in close proximity of the termination codon impaired NMD of the reporter mRNA. We also confirmed that this effect is NMD-specific and does not occur on mRNAs with the tethering sites downstream from the 3′ UTR, without direct binding of PABPC1, or when the termination codon is moved to a position downstream from the tethering sites. Notably, the siRNA-mediated depletion of UPF1 or UPF2 is epistatic to NMD suppression by PABPC1, demonstrating that PABPC1 acts in the same pathway as, but antagonistically to, UPF1 and UPF2. We also provide evidence that PABPC1 antagonizes the SMG6-dependent degradation pathway that initiates NMD by endocleavage in the vicinity of the termination codon. Since we have not investigated the role of other NMD factors, it will remain an important challenge for future studies to test whether the suppression of NMD affects additional degradation pathways, such as SMG5/SMG7-dependent deadenylation or mRNA decapping.

Although previous studies suggested that PABPC1 also antagonizes EJC-dependent NMD (Ivanov et al. 2008; Singh et al. 2008), in our experiments the NMD inhibition by PABPC1 is reduced in the presence of a downstream EJC. This suggests that the NMD of the reporter mRNA without 3′ UTR introns occurs in an EJC-independent manner and does not involve EJCs bound at noncanonical positions within the long 3′ UTR (Sauliere et al. 2012; Singh et al. 2012). We interpret the weak inhibition of EJC-dependent NMD by PABPC1 as a nonspecific effect that is comparable to the effects observed with unrelated reporter mRNAs. However, it is conceivable that the specific composition of EJCs determines their amenability to NMD suppression or that a subset of EJC components are inhibited by PABPC1. Hence, it remains to be determined whether PABPC1 is able to antagonize NMD activated by EJCs or other mRNA-bound proteins, or whether its function is restricted to long 3′ UTRs.

Current models of NMD suggest that a translation termination event in the proximity of the poly(A) tail prevents the interaction of UPF1 with eRF3a and therefore inhibits NMD, whereas translation termination upstream of a long 3′ UTR enables the association of UPF1 with eRF3a and activates NMD. In contrast to this model, we find that tethered PABPC1 does not require the interaction with eRF3a, but needs to bind to eIF4G to suppress NMD. Similar to PABPC1, tethered eIF4G also inhibits NMD. Additionally, mutants of eIF4G lacking the binding region for PABPC1 either by deletion or point mutation are still able to suppress NMD. These results suggest that binding of PABPC1 to eIF4G is at least partially dispensable for NMD suppression by eIF4G. We suggest that tethering of eIF4G downstream from the termination codon establishes a link of the site of translation termination to the 5′ end of the mRNA and facilitates ribosome recycling in a PABPC1-independent manner. Surprisingly, we also observe a strong suppression of NMD by tethered eRF3a. This result was unexpected in light of our PABPC1 results, which demonstrate that eRF3a is dispensable for NMD suppression by PABPC1. However, we hypothesize that tethering of eRF3a enhances ribosome recycling at the termination codon by the recruitment of PABPC1, which explains the inhibition of NMD by eRF3a. This hypothesis is supported by our observation that a mutant of eRF3a (F76A), which is unable to interact with PABPC1, is no longer able to inhibit NMD in the tethering assay. Of note, additional binding partners of PABPC1 known to compete with the interaction of eIF4G and eRF3a may be involved in the regulation of NMD suppression, but were not studied here. Furthermore, PABPC4 and eRF3b may confer tissue-specific NMD suppression comparable to their homologs PABPC1 and eRF3a, respectively (Chauvin et al. 2005; Burgess et al. 2011). In summary, we have started to map NMD-suppressing domains of eIF4G and eRF3a, but a precise identification of critical interactions will be required to delineate the network of proteins that contribute to NMD suppression.

**FIGURE 7.**
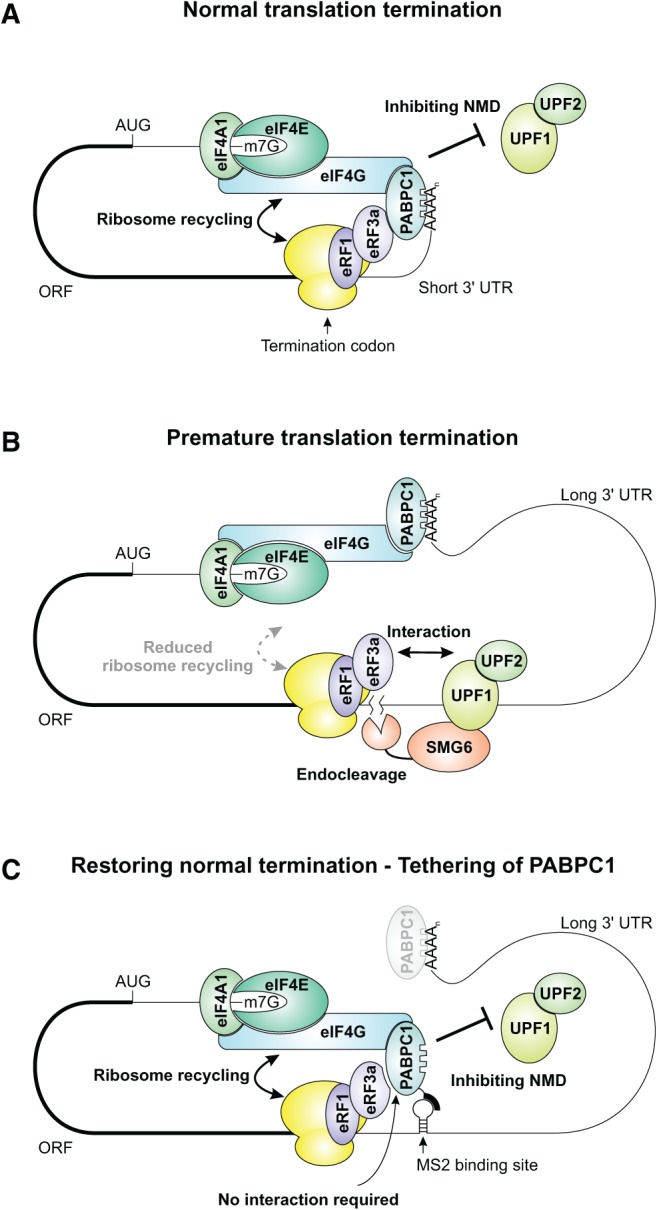
Model for a link between eIF4G-mediated ribosome recycling and NMD inhibition. (*A*) Normal translation termination and ribosome recycling of a short 3′ UTR-containing mRNA is enabled by PABPC1 interacting with both eRF3a and eIF4G, thereby preventing NMD. (*B*) Aberrant translation termination of long 3′ UTR-containing mRNAs activates NMD. The interaction of PABPC1 with eRF3a is decreased due to a large physical distance, preventing efficient ribosome recycling by eIF4G. Consequently, UPF1 is postulated to interact with eRF3a and elicit NMD. (*C*) Tethered PABPC1 inhibits NMD of a long 3′ UTR-containing substrate by bringing eIF4G in close proximity to the terminating ribosome. This proximity promotes a proper translation termination event and facilitates ribosome recycling, thereby antagonizing NMD activation.

The interaction of PABPC1 with eIF4G is thought to facilitate circularization of mRNAs, support ribosome recycling, and initiate translation (Wells et al. 1998; Kahvejian et al. 2005; Amrani et al. 2008). These processes, albeit being important for general translation, have not been previously linked to NMD. Hence, we suggest a revised model of NMD to include our findings (Fig. 7). According to this model, closed loop formation of the mRNA via PABPC1-eIF4G is important not only for translation initiation and ribosome recycling, but also for the suppression of NMD (Fig. 7A,C). We suggest that the interaction of PABPC1 with eRF3a establishes a branch connection from the site of translation termination to the 5′ end of the mRNA through the eIF4G-PABPC1 binding (Jackson et al. 2010). While the interaction of PABPC1 with eIF4G stimulates the removal of ribosomes from the site of termination, the inability to remove a ribosome after termination serves as an NMD activating signal and enables the recruitment of the NMD machinery to the position of the termination codon (Fig. 7B). This is in line with previous reports implicating that PTCs differ from normal termination codons most likely in the rate of ribosome dissociation subsequent to peptide hydrolysis (Kervestin and Jacobson 2012). We propose that the inability to recycle ribosomes is a stochastic event that can occur during every round of translation termination and no difference whatsoever is expected between different modes of translation initiation (Durand and Lykke-Andersen 2013; Rufener and Muhlemann 2013). While it is conceivable that many aberrant mRNAs are efficiently recognized during the first termination event, the correct position of the termination codon will be monitored during every translation cycle and serves to eliminate mRNAs that once escaped decay. Hence, the continuous recycling of ribosomes via mRNA circularization at the termination codon may represent a main mechanism to prevent the degradation of mRNAs with short 3′ UTRs. The precise molecular signal that recruits the surveillance complex to the site of termination still remains to be determined. UPF1 likely represents the factor that directly interprets the signals of terminating ribosomes, either via a direct interaction with the ribosome (Min et al. 2013) or by communicating with the eukaryotic release factors (Ivanov et al. 2008; Singh et al. 2008). However, it is unclear how phosphorylation of UPF1 is activated in the absence of a downstream EJC (Kashima et al. 2006). While our work complements the current model of NMD with an important role of the eRF3a-PABPC1-eIF4G interaction, the molecular function of many additional factors involved in NMD, such as components of the EJC, still needs to be integrated.

## MATERIALS AND METHODS

### Plasmids constructs

Plasmid constructs β-globin, WT300+e3, pCI-FLAG, pCI-MS2V5, pCI-mVenus, pCI-TPI, and expression vectors for PABPC1 and eIF4G were described previously (Gehring et al. 2005, 2009; Hundsdoerfer et al. 2005; Ivanov et al. 2008; Steckelberg et al. 2012). The modification of pCI-TPI with four copies of binding sites for the heterologous probe used in Northern blot analysis, as well as the insertion of the SMG5 3′ UTR and 4MS2 binding sites in the 3′ UTR of TPI were described elsewhere (V Boehm, N Haberman, F Ottens, J Ule, and NH Gehring, in prep.). Using the same cassette cloning strategy, the MINX splicing cassette was introduced in the vectors (TPI-4MS2-MINX-SMG5, TPI-MINX-4MS2-SMG5). Deletion and point mutants of PABPC1, eRF3a and eIF4G were generated by PCR, cloned in the designated expression vector, and verified by sequencing.

### siRNA transfections

HeLa cells were grown in 6-cm plates and transiently transfected with 300 pmol siRNA using Lipofectamine RNAiMAX (Life Technologies). At 24 h post-transfection the cells were transferred to 10-cm plates and 1 d later transfected again with 600 pmol siRNA. The following siRNA target sequences were used for Luciferase 5′-AACGTACGCGGAATACTTCGA-3′, for SMG6 5′-AAGGGTCACAGTGCTGAAGTA-3′, for UPF1 5′-AAGATGCAGTTCCGCTCCATT-3′, and for UPF2 5′-CACGTTGTGGATGGAGTGTTA-3′.

### Plasmid transfections

HeLa cells were grown in 6-well plates or transferred 1 d after siRNA transfection to 6-well plates and transfected by calcium phosphate precipitation with 0.3 µg of a mVenus expression plasmid, 0.5 µg control plasmid (β-globin or WT300+e3), and 2 µg plasmid encoding for reporter mRNA. For tethering or overexpression of tagged-protein, 0.8 µg of the FLAG- or MS2V5 expression plasmid was included in the transfection mix. For mRNA half-life experiments, cells were incubated with medium supplemented with 5 µg/mL Actinomycin D for 3 or 6 h prior to harvesting.

### RNA extraction and Northern blotting

Total RNA was extracted with Isol-RNA Lysis Reagent (5PRIME) and analyzed by Northern blotting as described (Gehring et al. 2009). Signals were quantified using a Typhoon Trio (GE Healthcare).

### Immunoblot analysis

SDS-PAGE and immunoblot analysis was performed using protein samples derived from Isol-RNA Lysis Reagent extractions. The antibodies against tubulin (T6074) and FLAG (F7425) were from Sigma, the antibody against V5 (18870) was from QED Bioscience, the antibodies against GFP (ab290) and SMG6 (ab87539) were from Abcam, and the antibodies against UPF1 and UPF2 were kindly provided by Jens Lykke-Andersen.

### Expression and purification of recombinant proteins

Strep-tagged PABPC1 wild type and mutants, GST-tagged PABPC1 1–496 and N-terminally GST-, C-terminally FLAG-tagged eRF3a and eIF4G constructs were expressed in *E. coli* Rosetta 2. Cells were grown to exponential phase in LB medium (OD_600_ = 0.6–0.8) and expression was induced with 0.2 mM IPTG overnight at 20°C. Strep-tagged proteins were purified via affinity chromatography using StrepTactin Superflow Plus columns (Qiagen). GST-tagged proteins were purified via affinity chromatography using GSTrap columns (GE Healthcare) followed by size exclusion chromatography using a Superdex 200 10/300 GL column (GE Healthcare). Cell lysis was performed in 40 mM Tris (pH 7.8), 250 mM NaCl with protease inhibitors (Protease inhibitor cocktail [Sigma], and 1 mM PMSF). All constructs were stored in 40 mM Tris (pH 7.8) and 150 mM NaCl.

### In vitro pull-down analysis

Three hundred picomoles of FLAG-tagged proteins (eIF4G 84-294 or eRF3a) were incubated with 250 pmol PABPC1 constructs in a final volume of 400 µL binding buffer (25 mM HEPES at pH 7.8; 150 mM NaCl; 2 mM MgCl_2_; 0.1% NP-40; 0.01% Triton X-100) in the presence of magnetic beads coupled to anti-FLAG antibodies (M2 magnetic beads; Sigma). After incubation for 2 h at 4°C, beads were washed twice with 500 µL wash buffer (25 mM HEPES at pH 7.8; 300 mM NaCl; 2 mM MgCl_2_; 0.1% NP-40; 0.2% Triton X-100) and coprecipitated proteins were eluted with 1x SDS loading buffer. 10% of the protein mix was used as input control, all samples were separated on 12% SDS–polyacrylamide gels and stained with Coomassie Brilliant Blue.

## References

[FATSCHERRNA044933C1] Amrani N, Ganesan R, Kervestin S, Mangus DA, Ghosh S, Jacobson A 2004 A faux 3′-UTR promotes aberrant termination and triggers nonsense-mediated mRNA decay. Nature 432: 112–118 15525991 10.1038/nature03060

[FATSCHERRNA044933C2] Amrani N, Ghosh S, Mangus DA, Jacobson A 2008 Translation factors promote the formation of two states of the closed-loop mRNP. Nature 453: 1276–1280 18496529 10.1038/nature06974 PMC2587346

[FATSCHERRNA044933C3] Behm-Ansmant I, Gatfield D, Rehwinkel J, Hilgers V, Izaurralde E 2007 A conserved role for cytoplasmic poly(A)-binding protein 1 (PABPC1) in nonsense-mediated mRNA decay. EMBO J 26: 1591–1601 17318186 10.1038/sj.emboj.7601588 PMC1829367

[FATSCHERRNA044933C4] Bhuvanagiri M, Schlitter AM, Hentze MW, Kulozik AE 2010 NMD: RNA biology meets human genetic medicine. Biochem J 430: 365–377 20795950 10.1042/BJ20100699

[FATSCHERRNA044933C5] Burgess HM, Richardson WA, Anderson RC, Salaun C, Graham SV, Gray NK 2011 Nuclear relocalisation of cytoplasmic poly(A)-binding proteins PABP1 and PABP4 in response to UV irradiation reveals mRNA-dependent export of metazoan PABPs. J Cell Sci 124: 3344–3355 21940797 10.1242/jcs.087692 PMC3178455

[FATSCHERRNA044933C6] Chang YF, Imam JS, Wilkinson MF 2007 The nonsense-mediated decay RNA surveillance pathway. Annu Rev Biochem 76: 51–74 17352659 10.1146/annurev.biochem.76.050106.093909

[FATSCHERRNA044933C7] Chauvin C, Salhi S, Le Goff C, Viranaicken W, Diop D, Jean-Jean O 2005 Involvement of human release factors eRF3a and eRF3b in translation termination and regulation of the termination complex formation. Mol Cell Biol 25: 5801–5811 15987998 10.1128/MCB.25.14.5801-5811.2005 PMC1168810

[FATSCHERRNA044933C8] Clerici M, Deniaud A, Boehm V, Gehring NH, Schaffitzel C, Cusack S 2013 Structural and functional analysis of the three MIF4G domains of nonsense-mediated decay factor UPF2. Nucleic Acids Res 42: 2673–2686 24271394 10.1093/nar/gkt1197 PMC3936715

[FATSCHERRNA044933C9] Durand S, Lykke-Andersen J 2013 Nonsense-mediated mRNA decay occurs during eIF4F-dependent translation in human cells. Nat Struct Mol Biol 20: 702–709 23665580 10.1038/nsmb.2575

[FATSCHERRNA044933C10] Eberle AB, Stalder L, Mathys H, Orozco RZ, Muhlemann O 2008 Posttranscriptional gene regulation by spatial rearrangement of the 3′ untranslated region. PLoS Biol 6: e92 18447580 10.1371/journal.pbio.0060092 PMC2689704

[FATSCHERRNA044933C11] Eberle AB, Lykke-Andersen S, Muhlemann O, Jensen TH 2009 SMG6 promotes endonucleolytic cleavage of nonsense mRNA in human cells. Nat Struct Mol Biol 16: 49–55 19060897 10.1038/nsmb.1530

[FATSCHERRNA044933C12] Gatfield D, Unterholzner L, Ciccarelli FD, Bork P, Izaurralde E 2003 Nonsense-mediated mRNA decay in *Drosophila*: at the intersection of the yeast and mammalian pathways. EMBO J 22: 3960–3970 12881430 10.1093/emboj/cdg371 PMC169044

[FATSCHERRNA044933C13] Gehring NH, Kunz JB, Neu-Yilik G, Breit S, Viegas MH, Hentze MW, Kulozik AE 2005 Exon-junction complex components specify distinct routes of nonsense-mediated mRNA decay with differential cofactor requirements. Mol Cell 20: 65–75 16209946 10.1016/j.molcel.2005.08.012

[FATSCHERRNA044933C14] Gehring NH, Lamprinaki S, Kulozik AE, Hentze MW 2009 Disassembly of exon junction complexes by PYM. Cell 137: 536–548 19410547 10.1016/j.cell.2009.02.042

[FATSCHERRNA044933C15] Glavan F, Behm-Ansmant I, Izaurralde E, Conti E 2006 Structures of the PIN domains of SMG6 and SMG5 reveal a nuclease within the mRNA surveillance complex. EMBO J 25: 5117–5125 17053788 10.1038/sj.emboj.7601377 PMC1630413

[FATSCHERRNA044933C16] Hoshino S, Imai M, Kobayashi T, Uchida N, Katada T 1999 The eukaryotic polypeptide chain releasing factor (eRF3/GSPT) carrying the translation termination signal to the 3′-poly(A) tail of mRNA. Direct association of erf3/GSPT with polyadenylate-binding protein. J Biol Chem 274: 16677–16680 10358005 10.1074/jbc.274.24.16677

[FATSCHERRNA044933C17] Hundsdoerfer P, Thoma C, Hentze MW 2005 Eukaryotic translation initiation factor 4GI and p97 promote cellular internal ribosome entry sequence-driven translation. Proc Natl Acad Sci 102: 13421–13426 16174738 10.1073/pnas.0506536102 PMC1224658

[FATSCHERRNA044933C18] Huntzinger E, Kashima I, Fauser M, Sauliere J, Izaurralde E 2008 SMG6 is the catalytic endonuclease that cleaves mRNAs containing nonsense codons in metazoan. RNA 14: 2609–2617 18974281 10.1261/rna.1386208 PMC2590965

[FATSCHERRNA044933C19] Hwang J, Maquat LE 2011 Nonsense-mediated mRNA decay (NMD) in animal embryogenesis: To die or not to die, that is the question. Curr Opin Genet Dev 21: 422–430 21550797 10.1016/j.gde.2011.03.008 PMC3150509

[FATSCHERRNA044933C20] Ivanov PV, Gehring NH, Kunz JB, Hentze MW, Kulozik AE 2008 Interactions between UPF1, eRFs, PABP and the exon junction complex suggest an integrated model for mammalian NMD pathways. EMBO J 27: 736–747 18256688 10.1038/emboj.2008.17 PMC2265754

[FATSCHERRNA044933C21] Jackson RJ, Hellen CU, Pestova TV 2010 The mechanism of eukaryotic translation initiation and principles of its regulation. Nat Rev Mol Cell Biol 11: 113–127 20094052 10.1038/nrm2838 PMC4461372

[FATSCHERRNA044933C22] Kahvejian A, Svitkin YV, Sukarieh R, M'Boutchou MN, Sonenberg N 2005 Mammalian poly(A)-binding protein is a eukaryotic translation initiation factor, which acts via multiple mechanisms. Genes Dev 19: 104–113 15630022 10.1101/gad.1262905 PMC540229

[FATSCHERRNA044933C23] Kashima I, Yamashita A, Izumi N, Kataoka N, Morishita R, Hoshino S, Ohno M, Dreyfuss G, Ohno S 2006 Binding of a novel SMG-1-Upf1-eRF1-eRF3 complex (SURF) to the exon junction complex triggers Upf1 phosphorylation and nonsense-mediated mRNA decay. Genes Dev 20: 355–367 16452507 10.1101/gad.1389006 PMC1361706

[FATSCHERRNA044933C24] Kervestin S, Jacobson A 2012 NMD: a multifaceted response to premature translational termination. Nat Rev Mol Cell Biol 13: 700–712 23072888 10.1038/nrm3454 PMC3970730

[FATSCHERRNA044933C25] Kononenko AV, Mitkevich VA, Atkinson GC, Tenson T, Dubovaya VI, Frolova LY, Makarov AA, Hauryliuk V 2010 GTP-dependent structural rearrangement of the eRF1:eRF3 complex and eRF3 sequence motifs essential for PABP binding. Nucleic Acids Res 38: 548–558 19906736 10.1093/nar/gkp908 PMC2811017

[FATSCHERRNA044933C26] Kozlov G, Gehring K 2010 Molecular basis of eRF3 recognition by the MLLE domain of poly(A)-binding protein. PLoS One 5: e10169 20418951 10.1371/journal.pone.0010169 PMC2854688

[FATSCHERRNA044933C27] Kozlov G, De Crescenzo G, Lim NS, Siddiqui N, Fantus D, Kahvejian A, Trempe JF, Elias D, Ekiel I, Sonenberg N, 2004 Structural basis of ligand recognition by PABC, a highly specific peptide-binding domain found in poly(A)-binding protein and a HECT ubiquitin ligase. EMBO J 23: 272–281 14685257 10.1038/sj.emboj.7600048 PMC1271756

[FATSCHERRNA044933C28] Loh B, Jonas S, Izaurralde E 2013 The SMG5-SMG7 heterodimer directly recruits the CCR4-NOT deadenylase complex to mRNAs containing nonsense codons via interaction with POP2. Genes Dev 27: 2125–2138 24115769 10.1101/gad.226951.113 PMC3850096

[FATSCHERRNA044933C29] Mendell JT, ap Rhys CM, Dietz HC 2002 Separable roles for rent1/hUpf1 in altered splicing and decay of nonsense transcripts. Science 298: 419–422 12228722 10.1126/science.1074428

[FATSCHERRNA044933C30] Min EE, Roy B, Amrani N, He F, Jacobson A 2013 Yeast Upf1 CH domain interacts with Rps26 of the 40S ribosomal subunit. RNA 19: 1105–1115 23801788 10.1261/rna.039396.113 PMC3708530

[FATSCHERRNA044933C31] Nicholson P, Muhlemann O 2010 Cutting the nonsense: the degradation of PTC-containing mRNAs. Biochem Soc Trans 38: 1615–1620 21118136 10.1042/BST0381615

[FATSCHERRNA044933C32] Nicholson P, Yepiskoposyan H, Metze S, Zamudio Orozco R, Kleinschmidt N, Muhlemann O 2010 Nonsense-mediated mRNA decay in human cells: mechanistic insights, functions beyond quality control and the double-life of NMD factors. Cell Mol Life Sci 67: 677–700 19859661 10.1007/s00018-009-0177-1 PMC11115722

[FATSCHERRNA044933C33] Okada-Katsuhata Y, Yamashita A, Kutsuzawa K, Izumi N, Hirahara F, Ohno S 2012 N- and C-terminal Upf1 phosphorylations create binding platforms for SMG-6 and SMG-5:SMG-7 during NMD. Nucleic Acids Res 40: 1251–1266 21965535 10.1093/nar/gkr791 PMC3273798

[FATSCHERRNA044933C34] Osawa M, Hosoda N, Nakanishi T, Uchida N, Kimura T, Imai S, Machiyama A, Katada T, Hoshino S, Shimada I 2012 Biological role of the two overlapping poly(A)-binding protein interacting motifs 2 (PAM2) of eukaryotic releasing factor eRF3 in mRNA decay. RNA 18: 1957–1967 23019593 10.1261/rna.035311.112 PMC3479387

[FATSCHERRNA044933C35] Rebbapragada I, Lykke-Andersen J 2009 Execution of nonsense-mediated mRNA decay: What defines a substrate? Curr Opin Cell Biol 21: 394–402 19359157 10.1016/j.ceb.2009.02.007

[FATSCHERRNA044933C36] Rufener SC, Muhlemann O 2013 eIF4E-bound mRNPs are substrates for nonsense-mediated mRNA decay in mammalian cells. Nat Struct Mol Biol 20: 710–717 23665581 10.1038/nsmb.2576

[FATSCHERRNA044933C37] Safaee N, Kozlov G, Noronha AM, Xie J, Wilds CJ, Gehring K 2012 Interdomain allostery promotes assembly of the poly(A) mRNA complex with PABP and eIF4G. Mol Cell 48: 375–386 23041282 10.1016/j.molcel.2012.09.001

[FATSCHERRNA044933C38] Sauliere J, Murigneux V, Wang Z, Marquenet E, Barbosa I, Le Tonqueze O, Audic Y, Paillard L, Roest Crollius H, Le Hir H 2012 CLIP-seq of eIF4AIII reveals transcriptome-wide mapping of the human exon junction complex. Nat Struct Mol Biol 19: 1124–1131 23085716 10.1038/nsmb.2420

[FATSCHERRNA044933C39] Schweingruber C, Rufener SC, Zund D, Yamashita A, Muhlemann O 2013 Nonsense-mediated mRNA decay—mechanisms of substrate mRNA recognition and degradation in mammalian cells. Biochim Biophys Acta 1829: 612–623 23435113 10.1016/j.bbagrm.2013.02.005

[FATSCHERRNA044933C40] Silva AL, Ribeiro P, Inacio A, Liebhaber SA, Romao L 2008 Proximity of the poly(A)-binding protein to a premature termination codon inhibits mammalian nonsense-mediated mRNA decay. RNA 14: 563–576 18230761 10.1261/rna.815108 PMC2248256

[FATSCHERRNA044933C41] Singh G, Rebbapragada I, Lykke-Andersen J 2008 A competition between stimulators and antagonists of Upf complex recruitment governs human nonsense-mediated mRNA decay. PLoS Biol 6: e111 18447585 10.1371/journal.pbio.0060111 PMC2689706

[FATSCHERRNA044933C42] Singh G, Kucukural A, Cenik C, Leszyk JD, Shaffer SA, Weng Z, Moore MJ 2012 The cellular EJC interactome reveals higher-order mRNP structure and an EJC-SR protein nexus. Cell 151: 750–764 23084401 10.1016/j.cell.2012.10.007 PMC3522173

[FATSCHERRNA044933C43] Steckelberg AL, Boehm V, Gromadzka AM, Gehring NH 2012 CWC22 connects pre-mRNA splicing and exon junction complex assembly. Cell Rep 2: 454–461 22959432 10.1016/j.celrep.2012.08.017

[FATSCHERRNA044933C44] Tani H, Imamachi N, Salam KA, Mizutani R, Ijiri K, Irie T, Yada T, Suzuki Y, Akimitsu N 2012 Identification of hundreds of novel UPF1 target transcripts by direct determination of whole transcriptome stability. RNA Biol 9: 1370–1379 23064114 10.4161/rna.22360 PMC3597577

[FATSCHERRNA044933C45] Thermann R, Neu-Yilik G, Deters A, Frede U, Wehr K, Hagemeier C, Hentze MW, Kulozik AE 1998 Binary specification of nonsense codons by splicing and cytoplasmic translation. EMBO J 17: 3484–3494 9628884 10.1093/emboj/17.12.3484 PMC1170685

[FATSCHERRNA044933C46] Uchida N, Hoshino S, Imataka H, Sonenberg N, Katada T 2002 A novel role of the mammalian GSPT/eRF3 associating with poly(A)-binding protein in cap/poly(A)-dependent translation. J Biol Chem 277: 50286–50292 12381739 10.1074/jbc.M203029200

[FATSCHERRNA044933C47] Wakiyama M, Imataka H, Sonenberg N 2000 Interaction of eIF4G with poly(A)-binding protein stimulates translation and is critical for *Xenopus* oocyte maturation. Curr Biol 10: 1147–1150 10996799 10.1016/s0960-9822(00)00701-6

[FATSCHERRNA044933C48] Wells SE, Hillner PE, Vale RD, Sachs AB 1998 Circularization of mRNA by eukaryotic translation initiation factors. Mol Cell 2: 135–140 9702200 10.1016/s1097-2765(00)80122-7

[FATSCHERRNA044933C49] Yepiskoposyan H, Aeschimann F, Nilsson D, Okoniewski M, Muhlemann O 2011 Autoregulation of the nonsense-mediated mRNA decay pathway in human cells. RNA 17: 2108–2118 22028362 10.1261/rna.030247.111 PMC3222124

[FATSCHERRNA044933C50] Zhang J, Sun X, Qian Y, Maquat LE 1998 Intron function in the nonsense-mediated decay of β-globin mRNA: indications that pre-mRNA splicing in the nucleus can influence mRNA translation in the cytoplasm. RNA 4: 801–815 9671053 10.1017/s1355838298971849 PMC1369660

